# Rush Medical College

**Published:** 1878-02

**Authors:** J. H. Etheridge

**Affiliations:** Assissant Secretary; Chicago


### Rush Medical College.

CHICAGO, July 5, 1876.

The following Resolution, adopted to-day, by the Faculty of this College, contains an answer to questions relative to Homœpathy sent to this Institution by the Dean of the University of Michigan:

"*Resolved*, That the time and attendance of students upon Lectures of the Medical Department of the University of Michigan, up to, and including, the last regular session of that College, may be recognized as part of the requisites for graduation in this College; but such time and attendance shall not hereafter be accepted so long as the teaching of Homœpathy, in whole or in part, shall be included in the course of study at that Institution."

J. H. ETHERIDGE,
Assissant Secretary.

